# Pharmacological effects of gastrodin: Insight into neurological diseases and mechanism in ferroptosis and pyroptosis

**DOI:** 10.1002/ibra.12118

**Published:** 2023-07-13

**Authors:** Xue Zheng, Jing Li, Zhao‐Qiong Zhu

**Affiliations:** ^1^ Department of Anesthesiology Zunyi Matermal And Child Health Care Hospital Zunyi Guizhou China; ^2^ Department of Cardiothoracic Surgery University Medical Center Regensburg Regensburg Germany; ^3^ Department of Anesthesiology Affiliated Hospital of Zunyi Medical University Zunyi Guizhou China

**Keywords:** ferroptosis, gastrodin, pharmacological effect, pyroptosis

## Abstract

Gastrodin, as an effective monomer of gastrodia elata, plays a significant role in anti‐inflammatory, antioxidant, antiapoptosis, and other aspects. As the global aging process continues to intensify, diseases of the central nervous system, cardiovascular system, and immune system have brought serious economic and mental burdens to families and have become a major challenge for global public health resources. Many studies have proved that gastrodin may be a potential drug for neurological diseases and ischemic injury but its mechanism of action is still unclear. [Correction added on 19 February 2025, after first online publication: In the preceding sentence, “the treatment of various systemic diseases” has been corrected to “neurological diseases and ischemic injury” in this version.]. In this study, the pharmacological action of gastrodin and the possible mechanism of regulating ferroptosis and pyroptosis were reviewed to provide a new treatment and research direction for clinicians and researchers.

## Graphical abstract

Pharmacological effects of gastrodin include prevention and treatment of cognitive decline and reperfusion injuries, anticonvulsion, antiepilepsy, antidepressants, and analgesia, which are related to antiferroptosis and antipyroptosis.

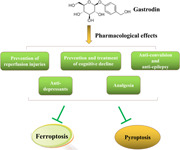

## 1 INTRODUCTION

Gastrodia elata has a long history of medical application. Middle‐aged and elderly people with cardiovascular and cerebrovascular diseases often use it as a functional food.^1^ Gastrodin is a phenolic glycoside derived from the rhizome of Gastrodia elata and is considered an indicator to measure the content of Gastrodia elata. Clinically, gastrodin mainly plays a role in anti‐inflammation, antioxidation, and antiapoptosis, maintaining glial cell homeostasis, promoting neural growth, and reducing ischemic brain injury.^2^, ^3^ In recent years, gastodin has found application in curing dementia, depression, Parkinson's disease, epilepsy, and migraines.^4^ Cellular death such as necroptosis, pyroptosis, autophagy, and ferroptosis is regulated by multiple factors and is a dynamic process that is tightly integrated with the occurrence and development of diseases. Cellular death is of great significance in biological processes such as body homeostasis and inhibition of the rapid proliferation of tumor cells.^5^ With the advancement of cellular death, the mechanism of gastrodin in the prevention and treatment of related diseases and its important role in the development of targeted therapy drugs have gradually gained due attention. This review summarized the research progress on the pharmacological effects of gastrodin, with a particular focus on its potential in neurological diseases and its underlying mechanisms involving ferroptosis and pyroptosis. [Correction added on 19 February 2025, after first online publication: The preceding sentence has been revised at the request of the author.].

## 2 PHARMACOLOGICAL EFFECTS OF GASTRODIN

### 2.1 Prevention and treatment of cognitive decline

Surging cases of cognitive dysfunction terribly increase the medical burden and downgrade the quality of life and working ability of patients. There are many theories for its pathogenesis, such as neuroinflammation, mitochondrial damage, β‐amyloid (Aβ), and elevated phosphorylated Tau protein, but only partial pathogenesis could be explained.^6^, ^7^, ^8^, ^9^ If the Alzheimer's disease (AD) model is established by injecting Aβ 1‐40 into the hippocampus of rats, gastrodin can improve their learning and memory abilities. Studies have shown that gastrodin can be used as a potential therapeutic drug for AD mainly triggered by neuroinflammation, which may be due to the improvement of cognitive function by inhibiting Aβ and its fibrous plaque.^10^ Zhao et al.^11^ found that gastrodin could temper the neurotoxicity of hippocampal neurons. It is achieved by upregulating the expression nuclear factor E2‐related factor 2 (Nrf2) and regulating the phosphorylation of kinase 1 and 2. The above results indicate that gastrodin may be a potential medicine for AD and other neurodegenerative diseases. Cognitive impairment is a very serious consequence of diabetes, but the underlying causes remain unknown. Cheng et al.^12^ found that gastrodin could improve the learning and memory ability of diabetic rats by improving Purkinje cell apoptosis and restoring the long‐term depression pathway. In conclusion, gastrodin has a good research prospect for the prevention and treatment of cognitive dysfunction‐related diseases.

### 2.2 Prevention of reperfusion injuries

When ischemic tissues have been resupplied blood for some time, the severity of damage will not alleviate but rapidly deteriorate. Such a condition is commonly defined as a reperfusion injury. Studies showed that^13^ after myocardial ischemia‐reperfusion injury (MIRI) when model rats are treated with gastrodin, the release of inflammatory factors interleukin‐6 (IL‐6) and tumor necrosis factor‐α decreases. Meanwhile, the uptake of Ca^2+^ in the sarcoplasmic reticulum increases so that calcium overload is lowered, thus improving MIRI. Zhang et al.^14^ have used gastrodin and rhynchophylline together to upregulate mir‐21‐5p and mir‐331‐5p in the ischemia‐reperfusion injury model. Therefore, the activation of inflammasomes is inhibited to protect nerves.

### 2.3 Antidepressants

Depressed behaviors are common in diabetic patients with diabetic encephalopathy. Lots of studies have concluded that^15^ endoplasmic reticulum stress and inflammatory response play an important role in the occurrence and progression of diabetic encephalopathy. Gastrodin has neuroprotective effects on the disease. The efficacy of improving depressive behaviors can be confirmed by forcing rats to undergo a swimming experiment. The mechanism may be related to attenuated endoplasmic reticulum stress and reduced activation of the nod‐like receptor 3 (NLRP3) inflammasome.

As the aging population keeps expanding, the incidence of stroke caused by hypertension, hyperlipidemia, and other related diseases is accordingly increasing. The common complication is poststroke depression, which is probably related to the damage of emotion‐regulatory neural circuits and the central nervous system (CNS).^16^ Li et al.^17^ have treated 78 patients with poststroke depression with gastrodin and fluoxetine hydrochloride. The results have shown that the depression scores of the patients treated with gastrodin are better than those of the control group. The levels of serotonin and neurotrophic factors were significantly higher than those of the control group, indicating that gastrodin can effectively relieve depression. The life quality of the patients has been greatly improved, which is worthy of promotion in clinical application.

### 2.4 Anticonvulsion and antiepilepsy

According to the World Health Organization, there are about 70 million epilepsy patients worldwide. The incidence is bimodal, with the highest rates in infants and the elderly. With multiple risk factors and a strong genetic predisposition, epilepsy is not a disease with a single expression and etiology.^18^ Yang et al.^19^ have found that gastrodin can enhance the expression of GABAA receptors through a rat model of temporal lobe epilepsy, thereby reducing the seizures and excitotoxicity of neurons. Currently, gastrodin exhibits the potential as an anticonvulsant. According to research reports on children's epilepsy, the lithium‐pilocarpine model is used to induce epilepsy in mice. After administration of gastrodin, electroencephalogram is recorded. Behaviors and brain‐derived neurotrophic factor (BDNF), nerve growth factor (NGF) levels, and the activity of adenosine 5'‐monophosphate‐activated protein kinase/peroxisome proliferator‐activated receptor‐α (AMPK/PPARα) are detected. The results show that gastrodin lowers the onset of pediatric epilepsy by inhibiting the levels of BDNF and NGF, and activating the AMPK/PPARα signal transduction pathway.^20^

### 2.5 Analgesia

Clinically, chronic pain can lead to mental problems such as anxiety and depression, which seriously reduces the quality of life. Chen et al.^21^ have confirmed that gastrodin has a significant analgesic property. In the chronic inflammatory pain model, after gastrodin treatment, it can be found that the mechanical and thermal pain domains are significantly improved. These studies further confirm that gastrodin has a strong analgesic effect. Wang et al.^22^ have found that gastrodin can relieve vincristine‐induced peripheral neuralgia. Its main mechanism may be associated with the regulation of NaV1.7 and NaV1.8 sodium channels. Cancer is a common disease that threatens human health. Chemotherapy‐induced peripheral neuropathy (CIPN) can disrupt patients' life. Qin et al.^23^ have used breast cancer cells and vincristine to establish a CIPN model. Gastrodin can restore the mechanical and thermal pain domains of rats without intervening with the antitumor effect of vincristine. This is achieved by inhibiting the inflammatory activation of microglia and the P38‐mitogen‐activated protein kinase signaling pathway, thus reducing the expression of inflammatory factors.

### 2.6 Other effects

Type 2 diabetes mellitus is a metabolic disease that seriously threatens people's life and health. It has been reported^24^ that gastrodin has an antidiabetic effect. Gastrodin promotes the phosphorylation of GATA‐binding protein 1 (GATA1) through the phosphatidylinositol 3 kinase/protein kinase B pathway and then enhances the transcriptional activity of GATA1. Next, the expression level of ubiquitin‐specific protease 4 increases, thereby lowering the ubiquitination and degradation of insulin receptors and improving insulin resistance.

Specifically, effective treatment for nonalcoholic steatohepatitis remains unavailable to date. Recently, it has been confirmed that gastrodin has a protective effect on various hepatic diseases. Wan et al.^25^ are fully convinced that gastrodin can improve nonalcoholic fatty liver by activating the AMPK signaling pathway through in vivo and in vitro experiments.

**Figure 1:**
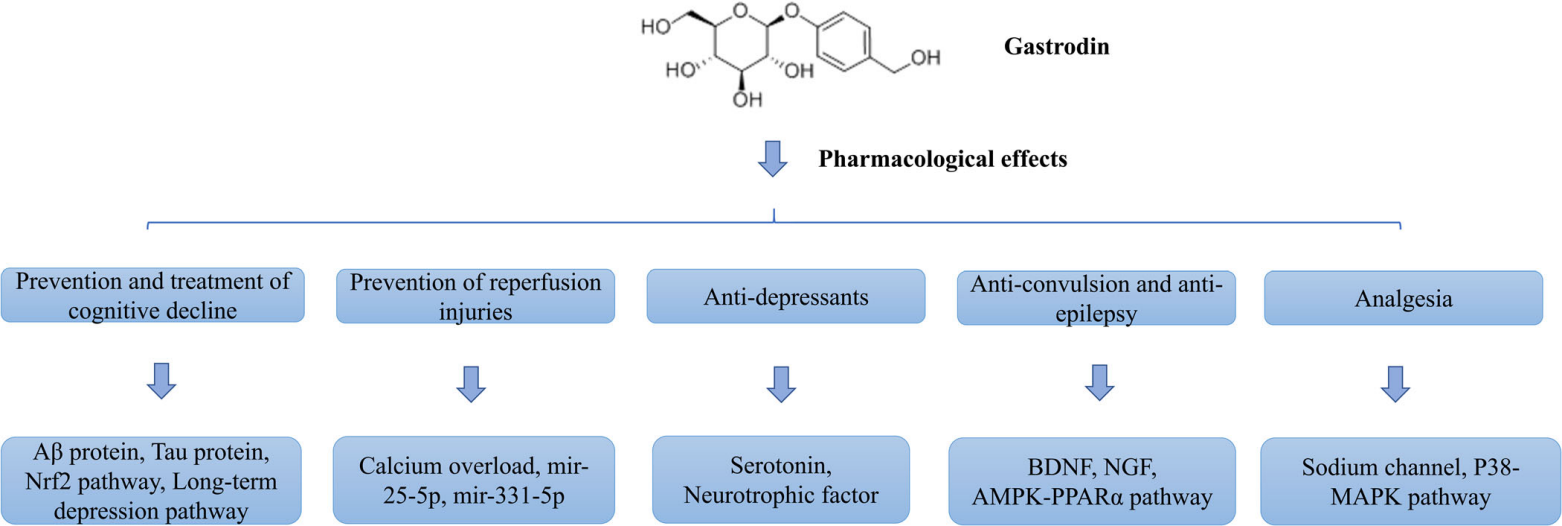
Pharmacological effects of gastrodin. [Color figure can be viewed at wileyonlinelibrary.com]

In summary, gastrodin plays a good role in improving cognitive function, preventing and treating reperfusion injury, antidepression, antiepilepsy, analgesia, and so on, which greatly expands the research and treatment field of gastrodin (Figure 1).

## 3 STUDY ON THE CORRELATION BETWEEN GASTRODIN AND FERROPTOSIS

### 3.1 Ferroptosis and its characteristics

Ferroptosis is a new type of programmed cellular death discovered in recent years, which is a nonapoptotic form dependent on iron ions.^26^ Studies have found that Erastin can specifically induce the death of Kirsten rat sarcoma viral oncogene cells. Ferroptosis does not trigger apoptotic bodies, activate the caspase family or break DNA.^27^, ^28^ However, it could be inhibited by iron chelators and activated by Ras‐selective lethal small molecule 3 (RSL3). In 2012, Dixon et al.^26^ defined this unique cellular death as ferroptosis.^29^

#### 3.1.1 Morphology of ferroptosis

Different from apoptosis, the ferroptosis structure of plasma membranes would disappear completely (Table 1). Cytoplasm and organelles would swell. Chromatin would accumulate. In addition, the morphological structure of mitochondria will also undergo significant changes. Main manifestations include a reduction in volume, a substantial increase in membrane density, rupture due to condensation of exterior membranes, and reduction or disappearance of cristae.^26^, ^30^

**Table 1:** Difference of four cell death.

	Ferroptosis	Pyroptosis	Apoptosis	Autophagy
Marker feature	The mitochondrial ridge reduces or disappears, the outer membrane breaks, wrinkles and the the mitochondria become deeply stained with color	The cells swell, the plasma membrane ruptures and pyrogenic bodies form	The chromatin condenses and breaks, the nucleolus disappears, nuclei shrink and become cracked and apoptotic bodies form	The autophagy lysosome forms
Other characteristics	The cell nucleus and cell membrane do not break	The cell breaks, the cytoplasmic component leaks	The cell is shrunk, the cytoplasm is leaked, the membrane‐bound vesicles form	The cell nucleus and cell membrane remain unchanged

#### 3.1.2 Biochemistry of ferroptosis

When large amounts of iron ions accumulate in the body, they peroxidate with lipid metabolism. Hence, ferroptosis is triggered by an abnormal increase of reactive oxygen species (ROS). During abnormal metabolism of anti‐oxidation, the accumulation of Fe^2+^ can induce the occurrence of a Fenton reaction to generate a large amount of ROS. Then, the ROS and polyunsaturated fatty acid (PUFA) on the surface of the cellular membrane undergo a peroxidative reaction. Next, the stability of the lipid bilayers is broken and the cellular membranes were lysed. Finally, ferroptosis occurs.^26^

### 3.2 Molecular mechanism of ferroptosis

Ferroptosis is a novel programmed cellular death. Its molecular mechanism is mainly closely related to intracellular glutathione (GSH) metabolism, lipid peroxidation, and iron metabolism.

#### 3.2.1 Amino acid metabolism and ferroptosis

The X_C_‐system is an amino acid reverse transporter protein in the cellular membrane. It is composed of SLC3A and SLC7A11. The X_C_‐system keeps cells working well by mediating extracellular cystine into the cytoplasm and transporting intracellular glutamate to the outside of the cell.^31^ All intracellular cystine can be synthesized into l‐cystine through oxidation. l‐cystine is synthesized by glutamic acid‐cysteine ligase and GSH enzymes and then is further converted to reduced GSH.^32^, ^33^ Glutathione peroxidase 4 (GPX4) is a selenium‐containing enzyme that converts potentially toxic lipid hydroperoxides into nontoxic lipid alcohols by reducing the synthesis of GSH to protect cells from oxidative damage after losing oxidative activity. Thereby, ferroptosis is inhibited. Erastin is the main agonist of ferroptosis. After the agonist combines with SLC7A11, it destroys the X_C_‐system. Erastin not only exhausts cysteine and GSH but also induces ferroptosis. The ferritin‐bound iron is released into the cytoplasm. RSL3 is another agonist of ferroptosis, which mainly binds covalently with GPX4 to inactivate GPX4.^34^ In addition, studies have found that FINO (2) can stimulate lipid peroxidation and indirectly inhibit the activity of GPX4, whereas DPI12 and cisplatin can deplete GSH to activate ferroptosis.^34^, ^35^, ^36^ Therefore, finding key molecules that regulate ferroptosis, such as GPX4 and GSH, is significant for studying various diseases that involve ferroptosis.

#### 3.2.2 Lipid metabolism and ferroptosis

The disorder of lipid metabolism is one of the pathogeneses of ferroptosis. PUFA, including arachidonic acids and docosahexaenoic acids, are the most susceptible to peroxidation during ferroptosis. When iron‐dependent ROS is excessively elevated, PUFA undergoes lipid peroxidation, leading to the damage of cellular membranes or iron‐dependent cellular death.^37^ Cyclooxygenase, cytochrome P450 and lipoxygenase are the most characterized lipid oxidases. However, lipoxygenase, instead of cyclooxygenase, has been shown to be involved in erastin‐mediated ferroptosis.^38^ In addition to lipoxygenases, some genes that regulate the synthesis of PUFA and maintain the integrity of normal cellular membranes may also affect the development of ferroptosis. Studies have shown that ^39^ recombinant lysophosphatidylcholine acyltransferase 3 (LPCAT3) and acyl‐CoA synthetase long‐chain family member 4 (ACSL4) may insert unsaturated arachidonic acids into the cellular phospholipid membrane to stimulate ferroptosis. In addition, Fe^2+^‐mediated Fenton reactions can also induce oxidative stress and lead to ferroptosis.^40^ Therefore, the activation of ferroptosis can be achieved by supplementing lipoxygenases and PUFA. Meanwhile, the reduction of ferroptosis can be achieved by inhibiting the activities of LPCAT3 and ACSL4.

Studies have shown that in the brains of patients with AD and mice with AD, ferroptosis‐related features such as disturbances in iron metabolism, glutamate excitotoxicity and deposition of lipid ROS can be detected.^41^ The accumulation of lipid ROS during ferroptosis can cause oxidative damage to cells, leading to neuronal damage and the development of AD.^42^ Previously, reperfusion is the most effective treatment for acute stroke. However, due to the increase of ROS and inflammatory factors, the damage to brain tissues was aggravated.^43^ In recent years, ferroptosis has been considered the cause of ischemia‐reperfusion injury. This view provides a new therapeutic approach to disease prevention and diagnosis. Several studies have shown that iron chelators can attenuate ischemic brain injury in animal models, exerting antioxidant and neuroprotective effects.^44^

#### 3.2.3 Iron metabolism and ferroptosis

Iron‐dependent lipid peroxidation is a key feature during the onset of ferroptosis. Under normal circumstances, human bodies absorb Fe^3+^ from animal meat, viscera, and other food. Fe^3+^ is reduced to Fe^2+^ under the action of duodenal pigment cells. Then, it is transported to epithelial cells of small intestines through the ferrous ion transporter 1 and absorbed.^45^ Metallic iron is crucial for the development of ferroptosis because it may generate ROS through Fenton reactions under physiological and clinical conditions. In the circulatory system, iron is transported to animal cells in the form of Fe^3+^ by transferrin (Tf) to keep cells functioning well. Tf can be recognized by Tf receptors on the cellular membranes and transferred to endosomes. In the acidic environment of the endosomes, Fe^3+^ is dissociated from the Tf receptor complex and converted to Fe^2+^ by the metal reductase (six‐transmembrane epithelial antigen of prostate 3). After reduction reactions, Fe^2+^ is transported by SLC11A2 to an iron pool in the cytoplasm.^46^, ^47^ On the one hand, under pathological conditions, the dynamic iron pool breaks the iron balance and leads to an imbalance in iron metabolism. Fe^2+^ oxidizes the lipids on the cellular membranes through Fenton reactions and generates lipid peroxides with PUFA under the catalysis of lipoxygenases, which breaks the normal structure of the cellular membranes and causes ferroptosis.^48^ On the other hand, as a cofactor of metabolic enzymes, Fe^2+^ can enhance the activity of metabolic enzymes, thereby promoting the generation of ROS.^49^

Therefore, related factors in the process of iron metabolism become potential targets for regulating ferroptosis. Studies have shown that the rate of ferroptosis induced by Erastin can be accelerated in the case of supplementing exogenous iron ions.^50^ Conversely, the administration of deferoxamine protects cells from Erastin‐ and RSL3‐mediated ferroptosis by sequestering intracellular iron.^51^ These results all indicate that the accumulation of iron ions is a unique mechanism to induce ferroptosis.

### 3.3 Gastrodin and ferroptosis

With the advancement of research, the regulatory mechanism of gastrodin to inhibit ferroptosis has been continuously improved. Mounting evidence show that gastrodin can temper the accumulation of ROS and the accumulation of iron ions in the process of ferroptosis. It is expected to become a new strategy for treatment. Inflammatory diseases are a series of diseases characterized by inflammatory responses, and ferroptosis is closely related to them.^52^ In tissues undergoing ferroptosis, there are some inflammatory factors related to ferroptosis metabolism.^53^ Studies have shown that ferroptosis and inflammatory diseases share similarities such as consumption of GSH, an increase of lipid peroxidation products and interruption of iron metabolism.^54^ In the study by Chen et al.,^21^ it has been confirmed that gastrodin significantly upregulates the expression of ferritin heavy chain 1 and GPX4, and reduces the expression of prostaglandin‐endoperoxide synthase 2. It suggests that gastrodin can resist ferroptosis by reducing lipid peroxidation.^21^ Gastrodin is the main functional substance of Gastrodia elata. It may become a functional food for the elderly because it can enhance resistance and delay ageing. Li et al.^55^ have confirmed that the gastrodin could improve cognitive dysfunction and the mechanism may be related to the activation of the Nrf2/Keap1‐GPx4 signaling pathway to inhibit the ferroptosis of hippocampal neurons, suggesting that Gastrodin may be a functional food for improving vascular dementia by inhibiting ferroptosis.

## 4 STUDY ON THE CORRELATION BETWEEN GASTRODIN AND PYROPTOSIS

### 4.1 Pyroptosis and its morphological characteristics

Pyroptosis, first proposed in 2001, is a pro‐inflammatory form of cellular death that depends on the regulation of cysteine protease‐1 after macrophages are infected with *Salmonella* or *Shigella*. Pyroptosis and apoptosis share partial morphological characteristics but also have many essential differences (Table 1). When pyroptosis occurs in the cell, the cell swells rapidly. Then, the plasma membrane ruptures. Next, small holes of 1–2 nm are formed. The permeability of the cellular membrane increases, so it loses the ability to allow substances to enter or exit the cell, thus resulting in a large amount of leakage of cytoplasmic components.^56^ At the same time, due to the influence of endonucleases and cysteine protease‐1, the DNA in chromosomes and chromatin is degraded.^57^

### 4.2 Molecular mechanisms of pyroptosis

#### 4.2.1 Classic pathway

This pathway is induced by caspase‐1‐mediated inflammasomes through microbial or nonmicrobial stimulation and is an important part of innate immunity.^58^ The inflammasome such as NLRP1, NLRP3, NLRC4, and AIM2 becomes the activation platform of cysteine protease‐1 after initiating and activating two pathways. Later, the precursor of caspase‐1 is pyrolyzed to an activated dimer. Then, the gasdermin family effector protein GSDMD is cut.^59^ The N‐terminal active domain of GSDMD aggregates to form pores on the cellular membrane. It acts as a mediator of pyroptosis and a direct passage to transport IL‐1β and IL‐18, ultimately causing pyroptosis and inflammatory responses.^60^ In addition, caspase‐1 can trigger pyrolysis of the precursor IL‐1β (pro‐IL‐1β) and precursor IL‐18 (pro‐IL‐18). Active IL‐1β and IL‐18 are released through the pores of GSDMD extracellularly to amplify the inflammatory responses.^61^

#### 4.2.2 Nonclassic pathway

This pathway is mainly mediated by caspase‐11, which belongs to the same pro‐inflammatory family as caspase‐1. Their sequences are different, so they play similar roles through different pathways. Caspase‐11 first binds the N‐terminal (caspase recruitment domain family member 8) CARD to lipopolysaccharide in Gram‐negative bacteria. Then, gasdermin domain‐containing protein (GSDMD) is pyrolyzed to N‐GSDMD. Finally, holes are formed on plasma membranes.^62^ However, caspase‐11 indirectly activates the NLRP3 inflammasome by pyrolyzing GSDMD, thereby initiating the NLRP3‐caspase‐1 pathway to generate IL‐1β and IL‐18.^63^ In addition, studies have found that caspase‐3 and caspase‐8 in the apoptosis family can also lead to the pyroptosis pathway by pyrolyzing gasdermin to release IL‐1β and IL‐18.^64^, ^65^

### 4.3 Gastrodin and pyroptosis

When some bacterial toxins and small dangerous molecules are sensed by extracellular receptors and delivered to cells, the assembly of corresponding inflammasomes, namely specific supramolecular complexes including NLRP1, NLRP3, NLRC4, AIM2, and pyrin, can be triggered.^66^ Among them, the NLRP3 inflammasome is most abundantly expressed in the CNS. It is involved in the regulation of many neuroinflammatory diseases. Therefore, the inflammatory responses generated by pyroptosis mediated by the NLRP3 inflammasome are inextricably correlated with the occurrence and development of neurological diseases.^67^ When a stroke occurs, the excessive activation of NLRP3 inflammasomes due to the efflux of K^+^ eventually induces pyroptosis. Unfortunately, the specific mechanism is still unclear.^68^ Studies have found that^69^, ^70^ infectious CNS diseases are related to the expression of caspase‐1. There is a typical pyroptosis characterized by an increase in the expression of IL‐1β and IL‐18. Caspase‐1 inhibitors are beneficial for treating diseases. In the future, pyroptosis will have an important impact on the treatment of nervous system‐related diseases.

Gastrodin has a significant therapeutic effect on the nervous system, but the mechanism of its protective effect on brain tissues in traumatic brain injury is unclear. Yang et al.^71^ have found that gastrodin can protect rats' brain tissues with traumatic brain injury by inhibiting the NLRP3 inflammasome signaling pathway. Hence, pyroptosis is prohibited. The injured nerves are recovered and the inflammatory responses are curbed. Gastrodin is a promising drug for treating traumatic brain injuries. In addition, gastrodin has been reported to inhibit the progression of stroke, but its regulatory mechanism is not fully elucidated. Zhang et al.^72^ have established a living cerebral ischemia/reperfusion injury model and have shown that gastrodin can significantly improve the nervous system score of rats and reduce the size of cerebral infarction. Meanwhile, gastrodin limits neuronal cell inflammation by regulating the IncRNA NEAT1/miR‐22‐3p axis, so the brain injuries caused by ischemia‐reperfusion are alleviated. In conclusion, gastrodin may become a new drug for curing cerebral ischemia‐reperfusion injuries.

## 5 LIMITATIONS AND PROSPECTS

This study reviews the research progress of gastrodin in regulating pyroptosis and ferroptosis, which will expand the research and treatment field of gastrodin, and has important practical significance for revitalizing local economic construction and creating good social effects. However, the role of gastrodin and its mechanism still have many puzzles to be verified, such as whether gastrodin can regulate ferroptosis or pyroptosis and improve the occurrence and development of cognitive dysfunction. It is believed that with the deepening of research, the role of gastrodin will gradually be widely recognized and applied in clinical practice.

## 6 CONCLUSION

**Figure 2:**
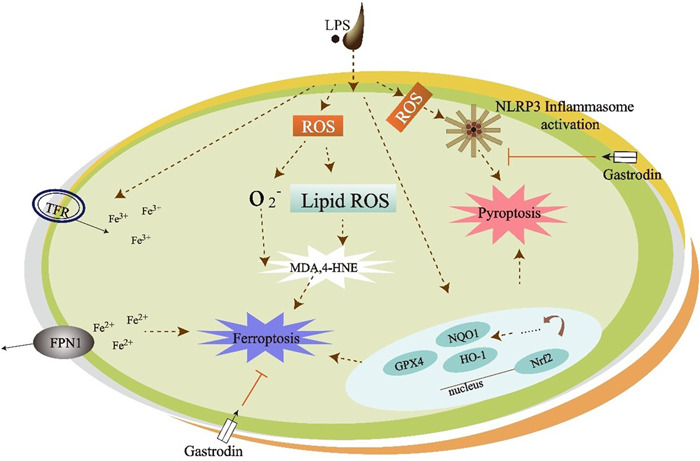
Mechanism diagram on antiferroptosis and antipyroptosis effects of gastrodin. [Color figure can be viewed at wileyonlinelibrary.com]

Gastrodin has significant preventive and therapeutic effects on related diseases in terms of preventing and treating cognitive decline, antidepression, and antiepilepsy. As the mechanism of therapies for different diseases may be the same or similar, it is easier for researchers to have a more comprehensive understanding of gastrodin monomers. By systematically elaborating the molecular mechanisms of ferroptosis and pyroptosis, as well as the relationship between gastrodin and ferroptosis and pyroptosis, we can see the possibility of gastrodin in curing dementia, depression, PD, epilepsy, migraine, and other diseases (Figure 2). However, many problems remain to be verified. It is believed that with further research, gastrodin will find broader applications in clinical practice.

## AUTHOR CONTRIBUTIONS

Xue Zheng collected materials and composed the paper, and Jing Li and Zhao‐Qiong Zhu modified the review.

## CONFLICT OF INTEREST STATEMENT

The authors declare no conflict of interest.

## ETHICS STATEMENT

Not applicable.

## Data Availability

Data sharing is not applicable to this article as no data sets were generated or analyzed during the current study.
